# Developmental Profile and Variability in Preschool‐Age Children With Down Syndrome

**DOI:** 10.1111/jir.70126

**Published:** 2026-06-02

**Authors:** Sara Onnivello, Giuseppe Ramacieri, Elisa Rossi, Maria Chiara Pelleri, Francesca Antonaros, Sara Colaianni, Chiara Marcolin, Francesca Pulina, Beatrice Vione, Francesca Catapano, Enrico Toffalini, Chiara Locatelli, Pierluigi Strippoli, Silvia Lanfranchi

**Affiliations:** ^1^ Department of Developmental Psychology and Socialization University of Padova Padova Italy; ^2^ Department of Medical and Surgical Science (DIMEC) University of Bologna Bologna Italy; ^3^ Specialist School of Child Neuropsychiatry University of Bologna Bologna Italy; ^4^ Department of Biomedical and Neuromotor Sciences (DIBINEM) University of Bologna Bologna Italy; ^5^ Department of General Psychology University of Padova Padova Italy; ^6^ Perinatal Comfort Care and Assistance to the Newborn with Congenital Malformations Unit, Department of Neonatology IRCCS Azienda Ospedaliero‐Universitaria di Bologna Bologna Italy

**Keywords:** developmental profile, Down syndrome, milestones, variability

## Abstract

**Background:**

The developmental and cognitive profile of Down syndrome (DS) has been studied across various age groups, but we have still a limited understanding of the preschool‐age range, including the domains that show the greatest variability at this age. Our study aims to (1) define the developmental profile in preschool‐age children with DS and (2) explore variability within domains of functioning.

**Method:**

We assessed 43 preschool‐aged children with DS (38–76 months) using the Griffiths‐III scales. This tool assesses development across five scales: Foundations of Learning, Language and Communication, Eye and Hand Coordination, Personal–Social–Emotional and Gross Motor Skills. We employed multivariate linear models for analysis.

**Results:**

The analyses revealed a peaks‐and‐valleys profile, with higher scores in the Personal–Social–Emotional and Gross Motor domains compared to the Foundations of Learning, Language and Communication and Eye and Hand Coordination scales. Notably, high interindividual variability was observed in the Language and Communication and Personal–Social–Emotional domains.

**Conclusions:**

Our findings identify strengths and weaknesses in specific domains within the preschool‐aged range and show higher variability in the verbal and personal–social–emotional domains. All this information offers valuable insights for early intervention planning, supporting parents, caregivers, and stakeholders in promoting optimal development in children with DS.

## 1 Introduction

Down syndrome (DS) is the most common genetic cause of intellectual disability and is associated with a specific behavioural phenotype characterized by global developmental delay and domain‐specific strengths and weaknesses (Grieco et al. 2015). Language, particularly expressive skills, represents the area of greatest difficulty, whereas socialization and non‐verbal abilities are often described as relative strengths (Abbeduto et al. 2001; Roberts et al. 2007; Vicari 2006). Motor development is also delayed, largely due to hypotonia and ligamentous laxity, although milestones are typically achieved in sequence (Malak et al. 2015).

Although this provides a general overview, research has also examined developmental profiles at different ages, starting from the first months of life. For example, Fidler et al. (2019) found that 9‐month‐old infants with DS scored lower in receptive and expressive communication compared to cognitive, fine motor and gross motor domains. Moreover, Onnivello et al. (2023) reported that older infants (3–17 months) obtained higher scores in expressive than receptive communication and in fine motor compared to gross motor skills. These findings suggest that a developmental profile characterized by peaks and valleys emerges already in the first years of life. Conversely, in a study of school‐aged children (7–16 years), a more homogeneous profile across verbal and non‐verbal domains was observed (Onnivello et al. 2022). Such discrepancies may reflect the dynamic nature of the developmental profile in DS, which might change with age. However, a gap remains in our understanding of development in DS: Most research has focused on infancy or school age, leaving the preschool period relatively underexplored. Therefore, our first aim is to examine the developmental profile of preschool‐aged children with DS.

Marked variability has also been observed within developmental domains. Studies suggest that individuals with DS show greater variability compared to typically developing (TD) peers (Karmiloff‐Smith et al. 2016). Such variability has been documented both in infancy (Fidler et al. 2019; Onnivello et al. 2023) and in school age (Onnivello et al. 2022; Tsao and Kindelberger 2009), with some studies identifying heterogeneity within specific cognitive or developmental domains, such as language (Zampini and D'Odorico 2011). Building on these findings, the second aim of the present study is to examine interindividual variability across developmental domains in preschool‐aged children with DS to determine whether such variability is consistent across domains or more pronounced in specific areas. In particular, greater variability is expected in language and communication, consistent with previous studies reporting high heterogeneity in this domain across ages (Onnivello et al. 2022; Zampini and D'Odorico 2011).

Investigating the average developmental profile of preschool children with DS, and the variability across different scales, can provide parents, caregivers and clinicians with valuable insights into what to expect at this age and which areas may show greater variability, thereby supporting the planning of more tailored interventions.

## 2 Methods

### 2.1 Participants

Forty‐three Italian children with DS (M = 55.12 months, SD = 12.54; age range = 38–76 months; 30 males) took part in the study. They were recruited during the annual follow‐up at the Neonatology Unit at the St. Orsola‐Malpighi Polyclinic in Bologna, Italy. Inclusion criteria were confirmed diagnosis of DS and age between 3 years and 6 years 11 months. Regarding co‐occurring conditions, 9% (*n* = 4) were born preterm, 21% (*n* = 9) had hearing loss, 23% (*n* = 10) presented with a congenital heart defect, and 9% (*n* = 4) had obstructive sleep apnoea.

### 2.2 Measurements

#### 2.2.1 Griffiths‐III

All the children were assessed with the Griffiths‐III scales (Green et al. 2016; Italian ed. Lanfranchi et al. 2019), a standardized test to assess the child's global development. It is composed of five scales: Foundations of Learning (Scale A), Language and Communication (Scale B), Eye and Hand Coordination (Scale C), Personal–Social–Emotional (Scale D) and Gross Motor (Scale E). Foundations of Learning assesses cognitive development during early childhood, including aspects of thinking, memory and play. Language and Communication measures overall communication development, including expressive language, receptive language and communication skills. Eye and Hand Coordination considers fine motor skills, manual dexterity, bimanual coordination and visual perception skills. Personal–Social–Emotional measures skills relating to the child's developing sense of self and growing independence, interactions with others, adaptive behaviour and aspects of early emotional development. Finally, Gross Motor assesses postural control, balance and gross body coordination, among other abilities.

For most scales, children are required to complete structured tasks that are administered (e.g., block building and object naming), or they are observed by a trained examiner to identify specific behaviours (e.g., pointing, babbling and grasping). The assessment is play‐based and uses materials familiar to young children (e.g., blocks and dolls). An exception is the Personal–Social–Emotional scale, for which some items are directly administered, whereas others—those involving social behaviour with peers or adaptive behaviours (e.g., eating skills and sphincter control)—are assessed through caregiver report, as such competencies are difficult to observe directly within the assessment setting.

Although standardized for ages 0–6 years, the test was administered to children up to 6 years 11 months, as tasks were appropriate for their developmental level and sensitive to individual differences. The Griffiths have been used previously with children with DS within this age range (e.g., Locatelli et al. 2021; Onnivello et al. 2024) and remains informative in clinical settings even when children fall outside the normative range (Stroud and Green 2022).

Following a procedure well documented in the field (Locatelli et al. 2021; Onnivello et al. 2022, 2024; Toffalini et al. 2019), raw scores for each scale were converted into age‐equivalent (AE) scores using Italian norms. Standard scores or developmental quotients were not used because they frequently reached floor levels and were therefore less informative for describing developmental profiles and relative strengths and weaknesses (see Toffalini et al. 2019). In addition, standard score conversion was not possible for all participants, as the chronological age of some children exceeded the upper limit of the normative tables (72 months), with the oldest participants being 76 months of age.

### 2.3 Procedure

Children were assessed by a psychologist in a quiet room, and the assessment lasted approximately 90 min. Written informed consent was obtained from the parents/caregivers before assessment at the Department of Developmental Psychology, University of Padova (Italy).

All data collection was conducted under the approval of the independent Ethics Committee of the University Hospital St. Orsola‐Malpighi Polyclinic, Bologna (Italy) and was performed in accordance with the Declaration of Helsinki.

### 2.4 Data Analysis

We examined the developmental profile of the group with DS by comparing mean (M) scores and standard deviations (SDs) across the five Griffiths‐III domains (*Foundations of Learning, Language and Communication*, *Eye and Hand Coordination*, *Personal–Social–Emotional* and *Gross Motor*). A multivariate linear model was estimated using the brms package in R (Bürkner 2018; R Core Team 2024), with AE scores as dependent variables. As we were interested in comparing average values and SDs across scales, we entered no predictors in the model and just compared the intercepts (estimates of mean values) and sigma values (estimates of SDs). Comparing the intercepts allowed us to analyse the overall average profile, whereas comparing the sigma values provided insights into the variability between scales, highlighting which scale exhibited the greatest variability. The differences across scales were calculated as subtractions across the posterior distributions and reported along with their 95% Bayesian credible intervals (CI; this is analogous to the ‘confidence interval’ in frequentist estimation). A CI that did not include the value of 0 was interpreted as a reliable difference between scales.

## 3 Results

Descriptive statistics for each domain are reported in Table 1.

**TABLE 1:** Descriptive statistics for the Griffiths‐III scales.

	Age‐equivalent scores M (SD) [min–max]
A: Foundations of Learning	23.12 (7.83) [8–43]
B: Language and Communication	23.49 (10.26) [4–49]
C: Eye and Hand Coordination	23.47 (8.09) [8–38]
D: Personal–Social–Emotional	26.30 (10.87) [10–49]
E: Gross Motor Skills	25.49 (8.79) [9–50]

**FIGURE 1:**
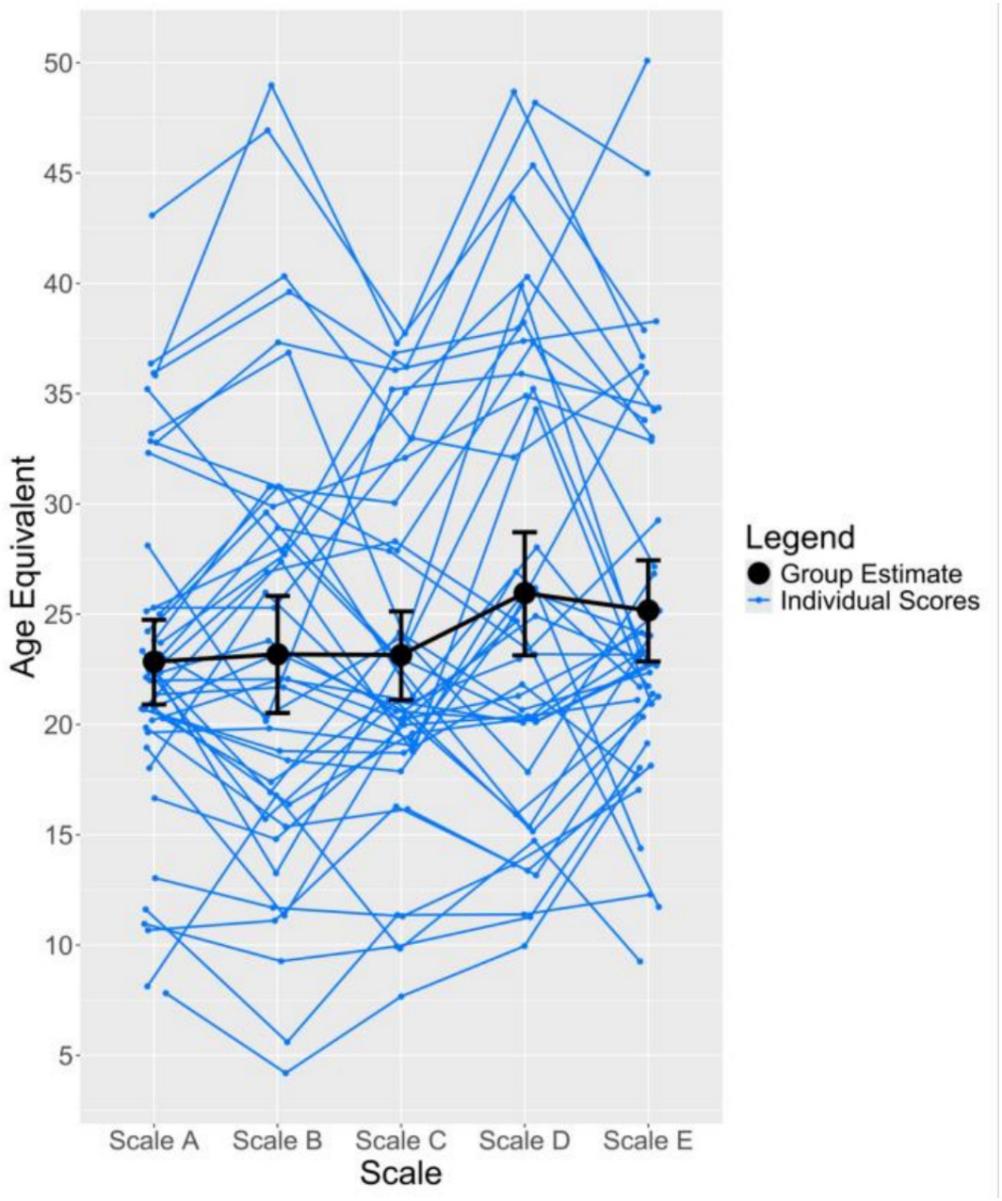
The Griffiths‐III age‐equivalent (months) profile. *Note:* Foundations of Learning (Scale A), Language and Communication (Scale B), Eye and Hand Coordination (Scale C), Personal–Social–Emotional (Scale D) and Gross Motor Skills (Scale E).

To define the developmental profile of children with DS, a multivariate linear model was run. The contrasts (reported in Table 2) were examined for both M and SDs of AE scores, which allowed us to define the average profile and difference in variability of scores between scales. The mean scores are graphically reported in Figure 1, along with the profiles of each individual.

**TABLE 2:** Contrast between scales—age‐equivalent scores.

	Mean estimated difference [CI]	SD estimated difference [CI]
Foundations of Learning		
Language and Communication	−0.35 [−2.03, 1.34]	**−2.40 [−3.89, −1.07]**
Eye and Hand Coordination	−0.32 [−1.61, 1.00]	−0.24 [−1.34, 0.87]
Personal–Social–Emotional	**−3.11 [−4.88, −1.33]**	**−2.84 [−4.44, −1.48]**
Gross Motor	**−2.32 [−4.08, −0.52]**	−1.14 [−2.71, 0.30]
Language and Communication		
Eye and Hand Coordination	0.04 [−1.98, 2.03]	**2.16 [0.56, 3.96]**
Personal–Social–Emotional	**−2.76 [−4.37, −1.17]**	−0.44 [−1.84, 1.01]
Gross Motor	−1.97 [−4.50, 0.65]	1.26 [−0.80, 3.41]
Eye and Hand Coordination		
Personal–Social–Emotional	**−2.80 [−4.66, −0.94]**	**−2.60 [−4.22, −1.08]**
Gross Motor	**−2.00 [−3.42, −0.58]**	−0.90 [−2.20, 0.30]
Personal–Social–Emotional		
Gross Motor	0.80 [−1.56, 3.18]	1.70 [−0.27, 3.66]

*Note:* CIs not including 0 indicate reliable differences between scales (bold values). Positive estimated mean (M) or SD differences indicate higher values for the scale reported on the left.

Considering the profile, there was evidence for higher scores in the *Personal–Social–Emotional* and *Gross Motor* domains. Specifically, *Personal–Social–Emotional* was higher than *Foundations of Learning*, *Language and Communication* and *Eye and Hand Coordination*, whereas *Gross Motor* was higher than *Foundations of Learning* and *Eye and Hand Coordination*. No significant differences were observed between *Foundations of Learning* and *Language and Communication*.

When analysing the variability between scales, greater variability with respect to the other scales emerged in *Language and Communication* and *Personal–Social–Emotional* domains. In particular, *Language and Communication* (SD = 10.26) showed higher variability than *Foundations of Learning* (SD = 7.83) and *Eye and Hand Coordination* (SD = 8.09). Meanwhile, *Personal–Social–Emotional* (SD = 10.87) skills exhibited greater variability than *Foundations of Learning* (SD = 7.83) and *Eye and Hand Coordination* (SD = 8.09).

## 4 Discussion

This study aimed to delineate the developmental profile of preschool children with DS and to examine interindividual variability across domains. In this sample, higher scores were observed in the Personal–Social–Emotional and Gross Motor scales compared to Foundations of Learning, Language and Communication and Eye and Hand Coordination, showing at this age a peaks‐and‐valleys profile. Moreover, larger interindividual variability was observed in the Language and Communication and Personal–Social–Emotional domains compared to the other areas.

Our results align with previous studies suggesting that the Personal–Social–Emotional domain represents an area of relative strength in individuals with DS compared to other developmental domains, both in preschool and school‐aged children (Fidler et al. 2006; Onnivello et al. 2023). However, although this domain emerged as a relative strength at the group level, our results also revealed marked interindividual variability within it. This pattern may suggest that social and emotional competencies, often described as characteristic strengths in the DS behavioural phenotype, are not uniformly preserved in preschool‐aged children. Such heterogeneity may reflect the interplay of multiple factors, including individual differences in temperament and cognitive resources, variability in parenting styles and educational environments and the extent of social experiences during early development. From a clinical perspective, these findings highlight the importance of individualized assessments rather than assuming consistently high socio‐emotional functioning across all children with DS. It is also important to note that in the present study, the Personal–Social–Emotional domain was assessed as a single composite scale, as defined by the Griffiths‐III. Although this provides a useful global index of social, emotional, personal and adaptive functioning, it does not allow differentiation of subdomains. Future studies should employ tools or methodologies that separately evaluate personal skills, autonomy, social interaction and emotional regulation to clarify whether all components represent consistent relative strengths or follow distinct developmental trajectories.

The high interindividual variability observed in the Personal–Social–Emotional domain may also be linked to the variability found in the Language and Communication domain. At this early stage, socio‐emotional engagement often relies heavily on emerging communicative skills, such as the use of gestures, vocalizations and early words to initiate and sustain interactions. Children with relatively stronger prelinguistic and language communication abilities may more effectively express needs, share emotions and engage in reciprocal interactions, whereas those with greater delays may show more challenges in adaptive and socio‐emotional behaviours. Future research should further explore the interplay between early communication and socio‐emotional development to inform targeted early interventions.

On the other hand, variability in the Language and Communication scale, which includes both expressive and receptive language skills, was expected and is consistent with previous studies reporting high heterogeneity in language development and vocabulary size during the first years of life (Fidler et al. 2019; Zampini and D'Odorico 2011). Individual factors such as hypotonia (Karimi and Nelson 2023), early hearing loss (Laws and Hall 2014), prematurity (Onnivello et al. 2023), congenital heart defects (Aoki et al. 2018; Visootsak et al. 2016) and reduced sleep efficiency (Arias‐Trejo et al. 2021; Edgin et al. 2015) may impact language acquisition. Environmental factors, including quantity and quality of caregiver linguistic input (Dulin et al. 2023) and access to early intervention or inclusive education, may likely contribute further to heterogeneity. The interaction between these environmental influences and the individual risk factors described above is likely to amplify heterogeneity, suggesting that language development in DS results from a complex interplay between biological vulnerabilities and the child's communicative environment.

Finally, the Gross Motor scale also emerged as an area of relative strength in our sample. Although motor development is typically delayed in children with DS due to hypotonia, ligamentous laxity and altered postural control (Malak et al. 2015), our findings suggest that, by the preschool years, many children achieve a degree of gross motor competence that appears relatively stronger compared to other developmental domains. One possible explanation is that, despite the delayed onset of motor milestones, these skills tend to follow a predictable developmental sequence and may benefit from consistent practice and early physiotherapeutic interventions. Moreover, gross motor abilities are less dependent on language and higher cognitive processes, which are more affected in DS, potentially contributing to their relative advantage.

This study has several limitations. The sample size was relatively modest, and the gender distribution was unbalanced, although this is consistent with the higher prevalence of males in DS (Ziemka‐Nalecz et al. 2023). As a result, the observed patterns may reflect characteristics of this specific cohort rather than those of the broader population of preschool children with DS; therefore, the findings should be interpreted with caution, and further studies are needed to generalize these results. Moreover, longitudinal studies are needed to examine whether the observed peaks and valleys remain stable over time and to explore how individual and environmental factors interact to shape developmental outcomes. Another limitation concerns the use of AE scores. Although their utility in reducing floor effects in populations with intellectual disability is well documented (e.g., Toffalini et al. 2019), it should be acknowledged that these scores are psychometrically less robust than standard scores.

Despite the limitations discussed above, the present study adds to the existing literature by providing data on a developmental period that has been relatively under‐represented in research on DS. Prior work has mainly focused on developmental profiles in infancy, when domain‐specific peaks and valleys are already observable (Fidler et al. 2019; Onnivello et al. 2023), or in school‐aged children, for whom profiles at the group level tend to appear more homogeneous across domains (Onnivello et al. 2022). Within this context, our results are consistent with previous findings showing lower performance in the verbal domain and relatively higher scores in the Personal–Social–Emotional domain (Fidler et al. 2006; Onnivello et al. 2023). However, our findings also revealed a relative strength in gross motor skills, an area that has previously been described as a weakness in younger children with DS (Fidler et al. 2006). Differences in developmental profiles across age groups are therefore not contradictory but may instead reflect the notion that DS is characterized by a dynamic rather than static developmental profile, with relative strengths and weaknesses that change over time (D'Souza and D'Souza 2022). In addition, the present study demonstrated marked interindividual variability in language and personal–social–emotional skills, consistent with previous evidence of substantial variability both in younger and older children with DS. Together, these findings help refine expectations regarding developmental outcomes during the preschool years, highlight variability as a characteristic feature of the syndrome and underscore the importance of designing individualized interventions that account for heterogeneous developmental trajectories and address a broad range of competencies.

## Funding

This research was co‐funded by the Italian Complementary National Plan PNC‐I.1 ‘Research initiatives for innovative technologies and pathways in the health and welfare sector’ D.D. 931 of 06/06/2022, ‘DARE – DigitAl lifelong pRevEntion’ initiative, code PNC0000002. Funding for S.O. (CUP: B53C22006250001) and F.A. (CUP: B53C22006450001) derives from this project. Fellowships for F.P. and C.M. were funded by donations from the Fondazione Rosa Pristina. The fellowship for G.R. for the duration of the project was funded mainly by the Fondazione Umano Progresso. Donations from the Fondazione Umano Progresso and other donors supported the purchase of the hardware and software needed to conduct the research.

## Ethics Statement

All data collection was conducted under the approval of the independent Ethics Committee at the St. Orsola‐Malpighi Polyclinic and University Hospital (Bologna, Italy) and was performed in accordance with the Declaration of Helsinki.

## Conflicts of Interest

The authors declare no conflicts of interest.

## Data Availability

Data are available upon request.
